# Association of nefopam use with postoperative nausea and vomiting in gynecological patients receiving prophylactic ramosetron: A retrospective study

**DOI:** 10.1371/journal.pone.0199930

**Published:** 2018-06-28

**Authors:** Sun-Kyung Park, Seokha Yoo, Won Ho Kim, Young-Jin Lim, Jae-Hyon Bahk, Jin-Tae Kim

**Affiliations:** Department of Anesthesiology and Pain Medicine, Seoul National University Hospital, Seoul, Republic of Korea; International University of Health and Welfare School of Medicine, JAPAN

## Abstract

**Background:**

Postoperative nausea and vomiting (PONV) is a common adverse effect of opioid-based intravenous patient-controlled analgesia (IV PCA). Nefopam has been considered as a good candidate for inclusion in multimodal analgesia because of its opioid sparing effect, but it can be emetic. This study aims to examine whether the use of nefopam combined with fentanyl in IV PCA was associated with the higher incidence of PONV in patients receiving prophylactic ramosetron after gynecological surgery.

**Methods:**

Data from 296 patients who underwent gynecological surgery were retrospectively reviewed. The patients received IV PCA containing either fentanyl 1500 μg and ketorolac 90 mg (Group K) or fentanyl 1500 μg and nefopam 80 mg (Group N). All patients in both groups received 0.3 mg of ramosetron at the end of surgery. The primary outcome measure was the incidence of PONV during the 3-day postoperative period.

**Results:**

No difference was observed in the incidence of PONV during the 3-day postoperative period between the two groups. However, the incidence of nausea on postoperative day 2 was significantly higher in Group N (10.3%) than in Group K (2.8%) (*P* = 0.016). Multivariable logistic regression analysis showed that the use of nefopam was not associated with a higher incidence of PONV (adjusted odds ratio, 1.616; 95% confidence interval, 0.952–2.743, *P* = 0.076). There were no differences in postoperative pain scores between the two groups.

**Conclusion:**

The combined use of nefopam with fentanyl in IV PCA was not associated with the higher incidence of PONV compared with the use of ketorolac and fentanyl combination in patients who received ramosetron as PONV prophylactic agent. However, prospective trials are required for a confirmative conclusion.

## Introduction

Postoperative analgesia is important to achieve patient rehabilitation and satisfaction.[1] Intravenous patient-controlled analgesia (IV PCA) is widely used in patients undergoing surgery with moderate to severe postoperative pain.[2] Opioids play a pivotal role in the management of postoperative pain with IV PCA,[3,4] and fentanyl is a commonly used opioid in IV PCA, providing potent analgesia. However, fentanyl is associated with a high incidence of postoperative nausea and vomiting (PONV), which varies from 20% to 60% according to the literature.[5] PONV is related to undesirable consequences, such as pulmonary aspiration, dehiscence of surgical wounds, dissatisfaction of patients, and delayed recovery.[6,7] As opioids alone require a high dose to achieve sufficient analgesia and can cause undesirable side effects, multimodal or balanced analgesia has been suggested.[8] The basic concept for multimodal analgesia is that by using analgesic agents with different mechanisms of action in combination, dosage and side effects of each medicine can be minimized.[8]

Nefopam, a centrally acting non-opioid analgesic drug, has emerged as a good candidate for inclusion in multimodal analgesia.[9] Nefopam exerts anti-nociceptive effects by inhibiting the synaptosomal reuptake of serotonin, dopamine, and norepinephrine.[4,9–11] Nefopam has been reported to be effective in postoperative pain control with an opioid-sparing effect.[1,9] However, nefopam itself can induce PONV according to some studies,[10,12] and there is relatively little information on the effect of nefopam on PONV when used in combination with fentanyl.[13–15]

Ramosetron, a selective 5-HT_3_ receptor antagonist, is widely used for the prevention and treatment of PONV. A previous trial showed that ramosetron was more effective than ondansetron in preventing vomiting and decreasing nausea related to fentanyl-based IV PCA. [16] However, no study has reported the use of ramosetron with nefopam-containing IV PCA. Considering that the antinociceptive effect of nefopam involves the inhibition of the synaptosomal reuptake of serotonin, the combination of ramosetron and nefopam can theoretically manifest in a mutually contrasting effect on 5-HT_3_ receptors. In that scenario, the antagonistic interaction between nefopam and ramosetron might attenuate the analgesic effects of nefopam or antiemetic effect of ramosetron. Although a previous study revealed that concomitant use of ondansetron and nefopam had no antagonistic interaction, [17] no study has evaluated the clinical consequence when ramosetron and nefopam are used concurrently.

Therefore, the aim of our study was to examine whether nefopam was associated with the higher incidence of PONV in gynecological patients who received ramosetron for PONV prophylaxis. To achieve this aim, we conducted a retrospective cohort study of the patients who used IV PCA containing fentanyl either with or without nefopam after gynecological surgery.

## Materials and methods

### Study design

Ethical approval for this study [IRB No. H-1610-056-798] was obtained from the Institutional Review Board of Seoul National University Hospital, Seoul, Korea. Written informed consent was waived because of the retrospective nature of the study. Because all data in the present study were obtained retrospectively from electronic medical records, the study was not publicly registered before collecting data. Our retrospective observational study is compliant to STROBE checklist (S1 Checklist). We reviewed the electronic medical records of 296 patients undergoing gynecological surgery between April 25, 2016 and July 4, 2016. We enrolled all patients who met the eligible criteria during the study period. Inclusion criteria were adult patients (age ≥ 18 years) who had used IV PCA after gynecological surgery under general anesthesia during the study period. Gynecological surgeries included laparoscopic ovarian cystectomy, laparoscopic salpingo-oophorectomy, laparoscopic hysterectomy, laparoscopic myomectomy, total abdominal hysterectomy, abdominal myomectomy, and vaginal hysterectomy. The exclusion criteria were patients with inadequate medical records, those who had maintained their use of IV PCA for less than 2 days, and those with a hospital stay of less than 2 days.

### PCA protocol

The regimen of IV PCA in the present study was not determined for research purposes and the analysis was performed retrospectively. Before May 29, 2016, IV PCA in our institution consisted of fentanyl and ketorolac. On May 30, 2016, the IV PCA regime was changed to nefopam and fentanyl. As the potency of nefopam 20mg lies in the range of morphine 6–12 mg according to previous literature,[10,18–21] we assumed that nefopam 20mg was equipotent with morphine 9 mg. Because ketorolac 30 mg was reported to equipotent with morphine 12 mg, [22] and the regimen was determined based on the assumption that the analgesic potency of nefopam 80 mg was equipotent to that of ketorolac 90 mg. The regimen of IV PCA before and after the change is described in Table 1. Patients who used IV PCA, including nefopam were labeled as Group N (nefopam group), and those who used IV PCA without nefopam were labeled as Group K (ketorolac group). The dosage of fentanyl was same in both groups. For all patients, the IV PCA was set to provide a continuous infusion of 1 mL/h, 1 mL bolus, and 15 minutes of lockout time. Every patient received 0.3 mg IV of ramosetron at the end of surgery for PONV prophylaxis according to the PONV guideline.[23] Rescue antiemetic treatment (ondansetron 4 mg IV) was additionally provided at the discretion of the attending physicians in response to vomiting or severe nausea or patient’s request. Ketorolac 30 mg IV or tramadol 100 mg IV was provided as rescue analgesics at the discretion of the attending physicians.

**Table 1:** The regimen of intravenous patient-controlled analgesia.

	Group N	Group K
IV PCA drugs	nefopam 80 mg (0.8 mg/ml) fentanyl 1500 μg (15 μg/ml) normal saline 62 ml	ketoroloac 90 mg (0.9 mg/ml) fentanyl 1500 μg (15 μg/ml) normal saline 67 ml
A total volume	100 ml	100 ml

IV PCA, intravenous patient-controlled analgesia; Group N, nefopam group; Group K, ketorolac group.

1 mL/h-continuous infusion; 1 mL-bolus; 15 minutes-lockout time.

Ramosetron 0.3 mg was administered at the end of surgery.

### Outcomes

The primary endpoint was the incidence of PONV during the 3-day postoperative period. Secondary outcomes included the incidences of PONV on the operation day, postoperative day (POD) 1, and POD 2, and pain scores assessed by a numeric rating scale (NRS; 0 = no pain, 10 = maximum pain imaginable) at the same time points, the median NRS score during the 3-day postoperative period, the occurrence of clamping of IV PCA during the 3-day postoperative period, the number of patients who received antiemetic agents during the 3-day postoperative period, and the timing of antiemetic agents were administered. Data on the administration of rescue analgesic agents were also collected. All outcome measures were routinely checked by ward nurses. The ward nurses performed the regular assessments on whether the patient suffered from nausea or not every 6 hours, and assessed the severity degree of nausea using 3-point scale (mild, moderate, or severe). If the patient complained of nausea before the nurse’s regular assessment, it was also recorded. Demographic data, length of hospital stay, and baseline clinical parameters which were previously identified as risk factors for PONV, were included in our study. The factors included a history of PONV, nonsmoking status, history of motion sickness, history of migraine, duration of surgery, use of volatile anesthetics, laparoscopic surgery, and intraoperative opioid use. Digestive diseases before surgery were also investigated.

### Sample size determination

A clinically significant difference was considered to be a difference of 15% in the incidence of PONV. The sample size calculation was based on a preliminary survey conducted in our institution. The preliminary survey reported that the incidence of PONV during the 3-day postoperative period in gynecological patients was 30%. Thus, for α risk of 0.05 and β risk of 0.20, we needed to enroll at least 242 patients (121 patients per group) for testing two-sided equality (PASS software 2008 ver. 8.0.16; NCSS statistical software, Kaysville, UT, USA). We decided to include 290 patients to account for exclusions because of insufficient documentations. The calculated sample size was also validated for the multivariable logistic regression analysis according to the rule that outcome events should be ten per each independent predictor.[24] For this study, it was estimated that 266 patients or more are necessary to permit unbiased accommodation of eight or fewer predictive variables in a multivariable logistic regression model (under the estimated 30% incidence of PONV). In addition, under the observed 26.0% incidence of PONV in 296 patients (i.e., 77 cases of outcome events), it allowed us to include up to 7 variables in our multivariate model based on the rule of requiring 10 cases with the outcome of interest for every variable in the model.

### Statistical analysis

Categorical variables were reported as absolute number (n) and relative frequencies (%), and compared using the χ^2^ test or Fisher’s exact test according to their expected counts. Continuous variables were reported as mean (standard deviation) or median [interquartile range] and tested for their normal distribution by the Kolmogorov–Smirnov test. After the normality was verified, Student’s *t*-test was performed for the analysis. If the data did not follow a normal distribution, they were analyzed by the Mann–Whitney *U* test. The incidences of PONV, PCA clamping, and the request of antiemetics were analyzed using χ^2^ test or Fisher’s exact test. Pain scores were analyzed using two-way repeated measures ANOVA. Data were analyzed using the SPSS software version 23.0 (IBM Corp., Armonk, NY, USA).

We conducted the logistic regression analyses according to our aims described in the introduction section. Logistic regression models were used to identify risk factors for PONV within 3 days of surgery. Univariate logistic regression analysis was performed first to identify potential risk factors for PONV from clinical and demographic variables. Multivariable logistic regression with the backward Wald stepwise variable selection process was then performed to identify independent predictors for PONV using initial inclusion criteria of *P*<0.3 on the univariate analysis. We also included possible risk factors (age, use of volatile agents, operation time, and nonsmoking status) as variables for multivariable logistic regression. We selected these variables as potential predictors of PONV based on clinical knowledge and previous literature.[23] All results with a *P* value<0.05 with 2-tailed analyses were considered significant.

## Results

A total of 301 patients who underwent gynecological surgery under general anesthesia during the study period were initially identified by screening of the electronic medical records. Three patients were excluded because of early discharge (postoperative hospital stay less than 2 days), and 2 patients were excluded because they were pediatric (aged 13 and 15 respectively). As a result, 296 patients were included in final analysis. No differences were observed in baseline patient characteristics between group N and group K (Table 2). Length of hospital stay was similar between the groups. One patient in group K had previous history of migraine before surgery. No patient had previous history of motion sickness before surgery. One patient in group K had previous history of gastroesophageal reflux disease before surgery. Three patients (2 in group K, 1 in group N) had previous history of chronic gastritis before surgery.

**Table 2:** Characteristics of patients receiving intravenous patient-controlled analgesia containing either nefopam and fentanyl or ketorolac and fentanyl.

Characteristics	Group N (n = 147)	Group K (n = 149)	*P*-value
Female gender	147 (100%)	149 (100%)	1.000
Age, years	47.59 (14.0)	48.58 (13.7)	0.459
Height, cm	159.00 (5.6)	158.47 (5.7)	0.586
Weight, kg	61.17 (11.1)	59.51 (9.3)	0.214
BMI, kg cm^-2^	24.21 (4.3)	23.70 (3.5)	0.362
ASA I or II/ III	144/3 (98.0%/2.0%)	148/1 (99.3%/0.7%)	0.369
Operation time, min	116.40 (73.9)	121.79 (99.5)	0.807
Volatile / TIVA	56/91 (38.1%/61.9%)	48/101 (32.2%/67.8%)	0.289
Laparoscopic surgery	85 (57.8%)	97 (65.1%)	0.198
Nonsmoking status	143 (97.3%)	145 (97.3%)	1.000
History of PONV	9 (6.1%)	9 (6.0%)	1.000
Remifentanil consumption during surgery, μg	965.24 (697.7)	955.44 (683.8)	0.964
Length of hospital stay, days	6 [4–7]	5 [4–7.5]	0.632

Data presented as mean (SD), median [interquartile range] or n (%)

Group N, nefopam group; Group K, ketorolac group.

BMI: body mass index, TIVA: total intravenous anesthesia

**Fig 1:**
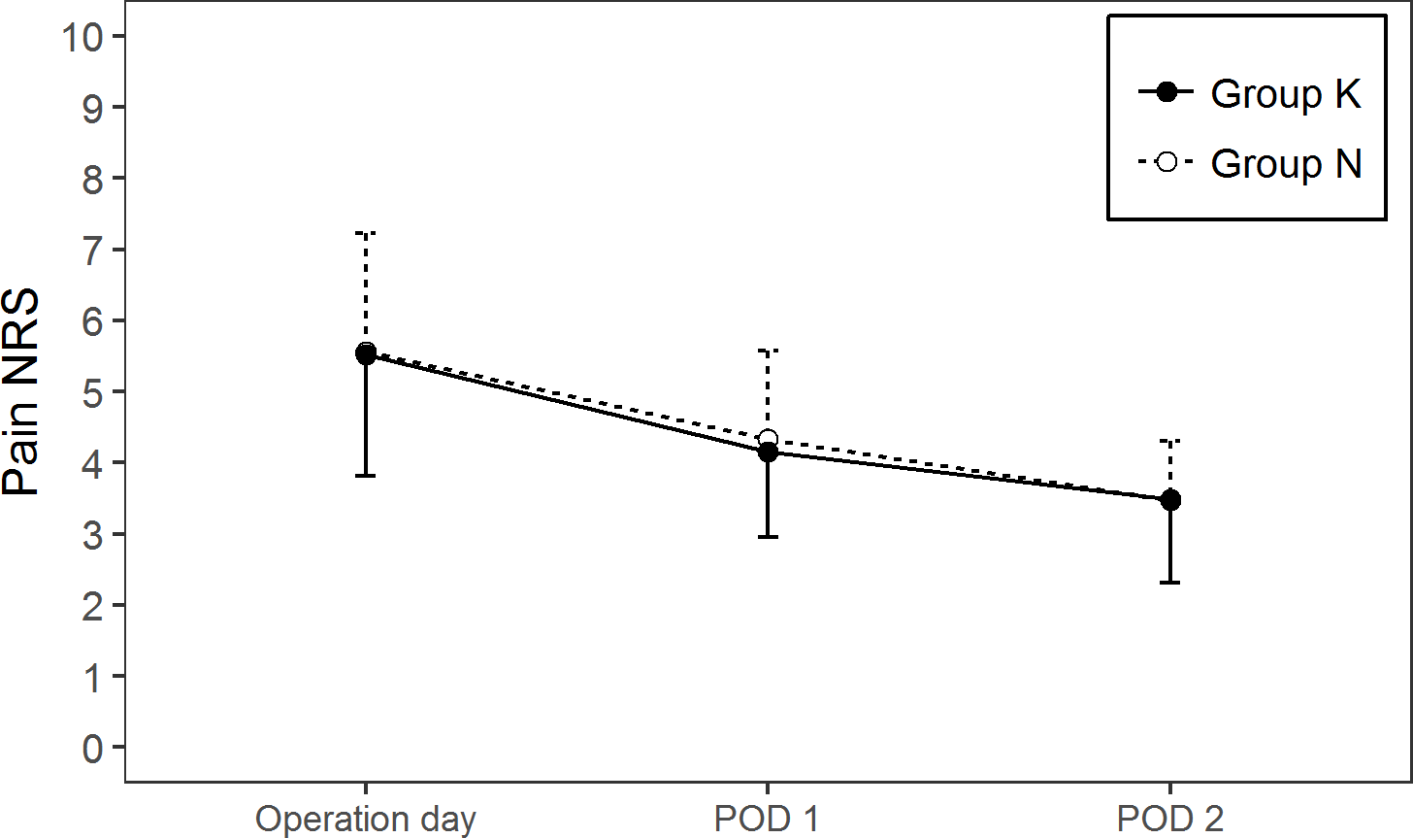
Numeric rating scale pain score assessed at different times during the 3-day postoperative follow-up period. Data are expressed as mean (SD). NRS, numeric rating scale; Group N, nefopam group; Group K, ketorolac group; POD 1, postoperative day 1; POD 2, postoperative day 2.

The overall incidence of PONV was 26.0% (n = 77). The incidence of PONV was not significantly different between group N (30.6%) and group K (21.5%, *P* = 0.073) (Table 3). However, the incidence of nausea on POD 2 was significantly higher in group N (10.3%) than in group K (2.8%, *P* = 0.016). There were no significant differences in the number of patients received rescue antiemetic agents between group N and group K on the operative day (16 patients (10.9%) vs. 11 patients (7.4%), respectively; *P* = 0.295), on POD 1 (11 patients (7.5%) vs. 7 patients (4.7%), respectively; *P* = 0.342), and on POD 2 (6 patients (4.1%) vs. 2 patients (1.3%), respectively; *P* = 0.172). There was no difference in severity of nausea between Group N and Group K at each day. Pain scores showed no differences between the two groups (Fig 1).

**Table 3:** The incidences of postoperative nausea and vomiting, and pain scores during the 3-day postoperative period.

	Group N (n = 147)	Group K (n = 149)	*P*-value
Nausea, op day	24 (16.3%)	18 (12.1%)	0.295
Vomiting, op day	0 (0%)	2 (1.3%)	0.498
Nausea, POD 1	26 (17.7%)	18 (12.1%)	0.175
Vomiting, POD 1	0 (0%)	1 (0.7%)	1.000
Nausea, POD 2*	15 (10.3%)	4 (2.8%)	0.016
Vomiting, POD 2*	1 (0.7%)	0 (0%)	1.000
Nausea, within 3 days	45 (30.6%)	32 (21.5%)	0.073
Vomiting, within 3 days	1 (0.7%)	3 (2.0%)	0.622
PONV, within 3 days	45 (30.6%)	32 (21.5%)	0.073
PCA clamping, within 3 days	55 (37.4%)	56 (37.6%)	0.976
Requirement of rescue antiemetic agents, within 3 days	28 (19.0%)	19 (12.8%)	0.138
Pain score, op day	5.572 (1.67)	5.545 (1.70)	0.882
Pain score, POD 1	4.352 (1.24)	4.159 (1.21)	0.204
Pain score, POD 2*	3.469 (0.83)	3.493 (1.18)	0.312
Median pain score, during 3-day postoperative period	4.262 (1.12)	4.193 (1.20)	0.449
Requirement of rescue analgesics, within 3 days	59 (40.1%)	56 (37.6%)	0.652
Dose of rescue analgesics administered within 3 days, morphine equivalent dose (mg)	8.03 (13.3)	6.88 (13.2)	0.280

Data are expressed as mean (SD) or number of patients (%).

Group N, nefopam group; Group K, ketorolac group; Op day, operation day, POD 1, postoperative day 1, POD 2, postoperative day 2. PONV, postoperative nausea and vomiting.

*For data on POD 2, 145 patients (Group N), and 144 patients (Group K) are included in the analysis, because of discontinuation of intravenous patient-controlled analgesia before outcome measurement on POD 2 (1 patient in Group N, 4 patients in Group K) and discharge from hospital before outcome measurement on POD 2 (1 in Group N, 1 in Group K).

Baseline patient characteristics according to the occurrence of PONV within 3 days of surgery are shown in Table 4. Although the patients who experienced PONV were slightly more likely to have used nefopam (58.4%) than those who did not experience PONV (46.6%), the difference was not significantly different (*P* = 0.073).

**Table 4:** Baseline patient characteristics by postoperative nausea and vomiting.

Characteristic	Total (n = 296)	Patients without PONV (n = 219)	Patients with PONV (n = 77)	*P*-value
Demographic data				
Age, years	48.09 (13.8)	48.49 (13.9)	47.82 (13.8)	0.880
Female, n	296 (100%)	219 (100%)	77 (100%)	1.000
Height, cm	158.74 (5.7)	158.78 (5.9)	158.61 (5.1)	0.787
Weight, kg	60.33 (10.2)	60.79 (10.3)	59.90 (10.2)	0.652
Body-mass index, kg m^-2^	23.96 (3.9)	24.01 (3.9)	23.81 (3.9)	0.602
ASA classification, n				0.999
I	146 (49.3%)	108 (49.3%)	38 (49.4%)	
II	146 (49.3%)	108 (49.3%)	38 (49.4%)	
III	4 (1.4%)	3 (1.4%)	1 (1.3%)	
Nonsmoking status, n	288 (97.3%)	213 (97.3%)	75 (97.4%)	1.000
History of PONV, n	18 (6.1%)	11 (5.0%)	7 (9.1%)	0.265
Surgery-related parameter				
Laparoscopic surgery, n	182 (61.5%)	134 (61.2%)	48 (62.3%)	0.858
Operation time, min	119.12 (87.6)	116.50 (89.9)	126.55 (80.7)	0.157
Anesthesia-related parameter				
Volatile anesthetics use, n	104 (35.1%)	75 (34.2%)	29 (37.7%)	0.589
Nefopam use, n	147 (49.7%)	102 (46.6%)	45 (58.4%)	0.073
Remifentanil consumption during surgery, μg	960.30 (689.6)	945.71 (688.3)	1001.82 (695.9)	0.482
Length of hospital stay, days	6 [4–7]	5 [4–7]	6 [5–8]	0.056

Values are expressed as mean (SD), median [interquartile range] or number (%). PONV: postoperative nausea and vomiting.

Table 5 shows the results of univariate and multivariable logistic regression analyses for PONV. No variable was significantly associated with PONV on univariable analysis. The use of nefopam and history of PONV were identified as candidate variables for multivariable analysis with a significance criterion of *P*<0.30. Multivariable analysis showed that the use of nefopam was not associated with PONV (adjusted odds ratio [OR], 1.616; 95% confidence interval [CI] 0.952–2.743, *P* = 0.076). No variable from our list of candidate variables was significantly associated with PONV on multivariable analysis.

**Table 5:** Multivariable analysis of patient characteristics associated with postoperative nausea and vomiting.

	Univariable Analysis		Multivariable Analysis	
Variable	Odds Ratio (95% CI)	*P*-value	Odds Ratio (95% CI)	*P*-value
Age, year	0.999 (0.979–1.018)	0.869	0.997 (0.978–1.017)	0.796
Nefopam use	1.656 (0.969–2.828)	0.065	1.616 (0.952–2.743)	0.076
Weight, kg	0.992 (0.966–1.019)	0.577		
Laparoscopic surgery	1.278 (0.703–2.324)	0.422		
Operation time, min	1.001 (0.997–1.005)	0.591	1.001 (0.998–1.004)	0.451
Volatile anesthetics	1.268 (0.668–2.408)	0.467	1.152 (0.668–1.987)	0.611
Nonsmoking status	1.026 (0.198–5.318)	0.975	1.088 (0.213–5.568)	0.919
History of PONV	1.738 (0.618–4.888)	0.295	1.783 (0.645–4.927)	0.265
Remifentanil consumption during surgery, μg	1.000 (1.000–1.001)	0.723		

CI = confidence interval

PONV = postoperative nausea and vomiting

## Discussion

Our retrospective study demonstrated that the combined use of nefopam with fentanyl in IV PCA did not increase the incidence of PONV after gynecological surgery in patients who received ramosetron. The incidence of PONV during the 3-day postoperative period after gynecological surgery was not significantly different between patients who received the nefopam–fentanyl combination and those who received the ketorolac–fentanyl combination. The use of nefopam was not a significant predictor for PONV after multivariable adjustment. Moreover, this is the first report showing that nefopam and ramosetron can be used in combination without any undesirable interaction.

Previous clinical trials with nefopam have reported inconsistent results on the incidence of PONV. Only a few previous randomized trials have found that nefopam reduced the incidence of PONV.[25,26] However, the incidence of PONV was often not reduced by opioid-sparing strategies with nefopam, as reported in several randomized trials.[1,13,14] Furthermore, a few studies have even showed a relatively frequent PONV incidence in patients treated with nefopam,[12,27] suggesting that nefopam is emetic. A previous systematic review showed nefopam was not significantly associated with PONV.[18] The present findings also suggested that nefopam is not significantly associated with PONV. However, our results should be cautiously interpreted considering the retrospective design.

A recent randomized clinical trial comparing nefopam and ketorolac as adjuvant analgesics for IV PCA reported that the incidence of PONV was higher in the nefopam group than in the ketorolac group.[27] However, the investigators did not use prophylactic antiemetics, which did not conform to the PONV guidelines since most patients included in the study had two or three major PONV risk factors. Considering the unusually high incidence of PONV (59%) in nefopam group in the study,[27] the results should be cautiously interpreted.

Although a large number of clinical trials have explored the use of nefopam in various surgical settings,[9] the efficacy of nefopam in PCA remains unclear.[17] Only a few studies have reported the efficacy and side effects of using nefopam combined with fentanyl in PCA,[13–15,27] as in the present study. Kim et al. compared three PCA groups after cardiac surgery: nefopam alone, fentanyl alone, or nefopam and fentanyl.[15] They found that pain scores were comparable between the groups and that the fentanyl-alone group had a significantly higher incidence of nausea. Moon et al. found that the combined use of nefopam and fentanyl in IV PCA significantly reduced fentanyl consumption after laparoscopic hysterectomy, but pain scores and the incidence of PONV was not significantly decreased in the nefopam group.[14] Jin et al. also showed that total PCA fentanyl consumption was reduced by the combined use of nefopam in PCA after laparotomy; however, the incidence of PONV showed no significant difference between the fentanyl and nefopam-fentanyl combination groups.[13] Our cohort study also showed that the combined use of nefopam with fentanyl in IV PCA after gynecological surgery resulted in comparable pain scores and no increase in the incidence of PONV compared with the ketorolac-fentanyl combination. Thus, the use of nefopam in combination with fentanyl in IV PCA may be a reasonable option for the management of moderate to severe postoperative pain. However, further research with these combining methods is required for a confirmative conclusion.

It is theoretically possible that the combination of nefopam and ramosetron leads to mutually contrasting modifications of serotonergic transmission mediated by 5-HT_3_ receptors, because ramosetron is a selective 5-HT_3_ receptor antagonist[28] and nefopam involves the inhibition of serotonin reuptake.[18,29] Although Lu et al. demonstrated that ondansetron did not attenuate the analgesic efficacy of nefopam,[17] there is no evidence that the combination of ramosetron and nefopam can be safely used without any undesirable interaction. To our knowledge, our study is the first to report the efficacy and safety of the co-administration of ramosetron and nefopam. Our findings may indicate that there is no antagonistic interaction between ramosetron and nefopam, suggesting that ramosetron can be used as antiemetics with nefopam, without compromising the analgesic efficacy of nefopam. However, prospective trials are required to validate our results.

The present study has several limitations. First, as our study had a retrospective design, there might have been an effect of unmeasured confounding variables. In particular, the administration of rescue antiemetic agents might cause potential bias in the incidence of PONV and it could not be completely controlled in the present study. However, to control potential confounding factors, we included only gynecological patients in our analysis, which was a relatively homogenous population in terms of PONV risk factors, and we also performed the multivariable logistic regression analysis. Second, our study data were derived from electronic medical records, which may have resulted in an underestimation of the true incidence of adverse events. In addition, potential risk factors for PONV, for example, the history of motion sickness, migraine, and digestive diseases, in patients included in our study might be possibly underestimated because of insufficient documentation. Third, we could not measure the cumulative consumption of IV PCA drugs because of retrospective design. Thus, we could not estimate the amount of each PCA drug consumed by each patient. Fourth, we could not obtain sufficient data about duration of PONV from our electronic medical records. Fifth, we could not blind all the participants to the kinds of used drug and could not exclude the possibility of unrecognized differences between the two groups as the present study retrospectively compared the PCA drugs before and after the selected time point. Lastly, considering the wide range of the confidence interval for the adjusted odds ratio, we could not eliminate the possibility that nefopam increases the risk of PONV.

In conclusion, our study demonstrates that the combined use of nefopam with fentanyl in IV PCA was not associated with a higher incidence of PONV compared to a ketorolac-fentanyl combination in gynecological patients receiving prophylactic ramosetron. Our findings suggest that nefopam in combination with ramosetron can be safely used via fentanyl-based IV PCA. However, prospective trials are required to validate our results.

## Supporting information

S1 Checklist A STROBE checklist for the present study. (DOC)

S1 File A dataset for the present study. (XLSX)
