# Correction: Association of nefopam use with postoperative nausea and vomiting in gynecological patients receiving prophylactic ramosetron: A retrospective study

**DOI:** 10.1371/journal.pone.0201084

**Published:** 2018-07-17

**Authors:** Sun-Kyung Park, Seokha Yoo, Won Ho Kim, Young-Jin Lim, Jae-Hyon Bahk, Jin-Tae Kim

There was an error in the affiliation for the authors. Sun-Kyung Park, Seokha Yoo, Won Ho Kim, Young-Jin Lim, Jae-Hyon Bahk, and Jin-Tae Kim are all affiliated with: Department of Anesthesiology and Pain Medicine, Seoul National University College of Medicine, Seoul National University Hospital, Seoul, Republic of Korea.
